# The Effect of Lurbinectedin as a Monotherapy and in Combination with Ionizing Radiation on Sarcoma Cell Lines

**DOI:** 10.3390/cancers17172930

**Published:** 2025-09-07

**Authors:** Hannah Alsheimer, Paula Schiller, Sabine Semrau, Felix Grabenbauer, Rainer Fietkau, Luitpold V. Distel, Laura S. Hildebrand

**Affiliations:** 1 Department of Radiation Oncology, Universitätsklinikum Erlangen, Friedrich-Alexander-Universität Erlangen-Nürnberg (FAU), Universitätsstraße 27, 91054 Erlangen, Germany; 2 Comprehensive Cancer Center Erlangen-EMN (CCC ER-EMN), 91054 Erlangen, Germany

**Keywords:** lurbinectedin, soft tissue sarcoma, radiotherapy, chemotherapy, clonogenic cell assay, scratch assay, ionizing radiation, radiation therapy

## Abstract

Background/Objectives: Soft tissue sarcomas are rare, heterogeneous tumors with limited therapeutic options and suboptimal outcomes in advanced stages. Lurbinectedin is a promising new antineoplastic alkylating agent. This study investigates its cytotoxic effects and its potential as a radiosensitizing agent on soft tissue sarcoma. Methods: Four soft tissue sarcoma cell lines were treated with lurbinectedin alone or in combination with ionizing radiation. Single-dose irradiation in a 4-day protocol was compared with prolonged treatment and an additional fractionated ionizing radiation scheme in a 6-day protocol. Cellular responses were analyzed by flow cytometry for apoptosis (Annexin V)/necrosis (7AAD) and cell cycle (Hoechst), clonogenic cell survival, and scratch assays for cell migration. Results: In the 4-day protocol, lurbinectedin induced G2/M arrest in all cell lines (*p* = 0.029) and significantly increased apoptosis/necrosis (*p* = 0.029) in SW-872. Lurbinectedin-treatment resulted in a decrease (*p* ≤ 0.002) of clonogenic cells in all cell lines. In the scratch assay, cell migration was delayed in two cell lines (*p* = 0.048) after lurbinectedin-treatment. Additional radiotherapy had no significant effect compared to lurbinectedin-monotherapy in apoptosis/necrosis and G/2M arrest in the 4-day protocol, clonogenic cell assay, and scratch assay. In the 6-day protocol, lurbinectedin induced an increase (*p* = 0.029) in G2/M arrest in all cell lines and apoptosis/necrosis in three cell lines, while resulting in a decrease (*p* < 0.001) of clonogenic cells. Additional radiotherapy had a significant effect on the decrease in clonogenic cells (*p* ≤ 0.048) in two cell lines but did not increase G2/M arrest and apoptosis/necrosis. Conclusions: Lurbinectedin had strong effects on three of the selected cell lines by inducing G2/M arrest, promoting apoptosis/necrosis, and reducing clonogenic survival, suggesting that it may be a promising chemotherapeutic agent in soft tissue sarcoma treatment. The effect on the fourth cell line was limited, as well as the effect on cell migration. Single-dose irradiation occasionally interfered with the effects of Lurbinectedin, whereas adding fractionated irradiation caused an additional decrease in clonogenic survival, indicating that the combination of Lurbinectedin with fractionated ionizing radiation may have promising effects.

## Simple Summary

Soft tissue sarcoma is a rare and heterogeneous group of tumors. Especially when metastasized, the treatment options remain limited. Therefore, this study evaluates the potential of Lurbinectedin, a chemotherapeutic agent, for the treatment of soft tissue sarcoma—both as a monotherapy and in combination with radiotherapy using four sarcoma cell lines. All cell lines showed a significant increase in G2/M arrest after treatment with Lurbinectedin. Three out of four cell lines showed an increase in apoptosis/necrosis after treatment with Lurbinectedin and all cell lines had reduced clonogenic survival. The combination of Lurbinectedin with fractionated ionizing radiation further decreased clonogenic survival. In the scratch assay, the cell migration of two out of four cell lines was reduced by Lurbinectedin. These findings suggest a broad sensitivity of sarcoma cell lines to Lurbinectedin and highlight its potential as a new chemotherapeutic agent, especially in a multimodal treatment plan.

## 1. Introduction

Sarcomas are tumors of mesenchymal origin, typically occurring in connective tissue [1]. Following the “WHO Classification of Tumors of Soft Tissue and Bone”, they can be differentiated into two major categories of soft tissue sarcomas (STS) and bone sarcomas, which can be divided further into over 50 different histological subtypes [2]. STS appear approximately four times more frequently than bone sarcomas [1,3]. Therefore, we focused on STS. Given the low incidence rate of STS of 5 cases per 100,000 inhabitants in Europe, they are classified as rare tumors [4]. STS are responsible for less than 1% of malignant tumors in the general population but represent around 8% of malignancies in adolescents and young adults [2,5]. Although they appear predominantly in the extremities, STS can also develop in organs and the cardiovascular system [1,5]. The five-year overall survival rate for STS is around 50% [6].

If possible, STS are treated surgically. However, due to delayed diagnosis, 18% of the patients already have distant metastases at initial diagnosis and over all up to 40% of patients with STS develop metastases, which limit the treatment options; metastatic disease is associated with a decreased five-year survival rate of 24.8% [7,8,9].

Radiotherapy (RT) is predominantly applied before surgery to enhance local control in resectable STS; however, RT also increases acute complication rates [10]. The response rate to RT is very variable depending on the different histological subtypes [11]. In the treatment of STS, RT is typically applied using a fractionated scheme of 2 Gy per fraction [12]. However, in the palliative setting, there is an increasing use of hypofractionated schemes with higher single-doses, such as 3 Gy per fraction, to achieve a more rapid symptom relief and to improve efficiency against radioresistant sarcoma subtypes [13,14]. Technological advancements have made RT more personalized, leading to improved treatment precision and better treatment outcome [15,16,17]. RT is now increasingly expanding to the management of unresectable tumors, either as a monotherapy or as part of a multimodal treatment strategy [16,17]. Especially the combination of RT with radiosensitizing agents could further improve therapeutic efficacy in STS therapy [17].

First-line chemotherapy (CT) for STS consists of the anthracycline Doxorubicin and the alkylating agent Ifosfamide [18,19,20], but in recent years there has been a shift towards incorporating additional agents tailored to the histological subtype of the patient-specific STS [21]. Despite the new treatment options, the outcome for patients with metastatic or recurrent STS remains suboptimal, especially due to the fact that only a part of the patients respond to those agents [22]. Furthermore, patients receiving RT and/or CT reported lower health-related quality of life compared to those not undergoing the treatments, indicating a negative impact on both physical and mental health values [23].

Lurbinectedin (LU) is an antineoplastic alkylating agent which inhibits active transcription and the repair system of the DNA in tumor cells [24,25]. It binds to the minor groove of DNA, particularly to CG-rich sequences situated near the promoter regions of protein-coding genes causing single-strand breaks and double-strand breaks which lead to cell cycle arrest and cell death [26]. Furthermore, it causes an irreversible degradation of the RNA Polymerase II, decreases circulating monocytes, reduces tumor infiltration of macrophages and diminishes the vascular density of the tumor [27,28].

LU is a structural analog of Trabectedin, which is already approved for the treatment of advanced STS and works well in combination with Doxorubicin [29,30,31]. However, early phase I/II studies suggested that with equivalent dosing schedules the maximal-tolerated dose of LU is over three times higher than that of Trabectedin [28]. In addition, compared to Trabectedin, LU has a lower plasma clearance (11.2 L/h/m^2^ vs. 39.98 L/h/m^2^) and a smaller distribution volume (438 L/m^2^ vs. 1460 L/m^2^), suggesting a longer lasting systemic exposure and a more restricted tissue distribution [32,33]. These pharmacokinetic differences could contribute to improved tolerability [34]. LU caused less nausea and vomiting when compared to Trabectedin. Grade 3–4 elevations in the liver enzymes alanine aminotransferase (5% vs. 26%) and aspartate aminotransferase (2% vs. 13%) occurred less frequently when using LU compared to Trabectedin which may have a particular relevance for patients with pre-existing liver dysfunction [35,36,37]. However, LU can cause myelosuppression, with Grade 3–4 leukopenia observed in 46% of patients, compared to 37% with Trabectedin which suggests that it may not be as suitable for patients with pre-existing hematologic conditions [35,36]. Still, neutropenia was largely manageable in clinical studies using LU with the majority of patients tolerating the standard dose of 3.2 mg/m^2^ without requiring modification [38,39].

Currently, LU is approved as a second line treatment for small lung cell cancer [40]. In some early-phase trials it was also tested on selected STS. LU showed activity in relapsed Ewing sarcomas and achieved a response rate of approximately 60% in leiomyosarcomas when combined with doxorubicin [41,42].

To investigate the potential of LU in STS treatment, we have selected four malignant STS cell lines of distinct different histological subtypes.

Fibrosarcomas are diagnosed by exclusion and can be further classified into subtypes such as myxofibrosarcoma and sclerosing epithelioid fibrosarcoma, all of which tend to show chemoresistance [43]. SW-684 is a fibrosarcoma cell line that has not been further histologically subclassified and is therefore generally referred to as fibrosarcoma. It exhibits a fibroblast-like growth pattern [44]. Since no subtype information is available, survival and metastasis data from the various fibrosarcoma subtypes cannot be directly applied. In previous in vitro studies, SW-684 has been characterized as a cell line that tends to be resistant to chemotherapeutic agents, consistent with the general chemoresistance reported in all fibrosarcoma subtypes [43]; therefore, it was included in this study as a representative cell line for chemoresistant STS [45,46].

Liposarcomas can be classified into four subtypes: myxoid and well differentiated liposarcomas, which are considered low-grade tumors, and pleomorphic and dedifferentiated liposarcomas, which demonstrate more aggressive behavior [47]. The five-year survival rate and metastatic recurrence are highly variable across the liposarcoma subtypes [48]. SW-872 is a cell line that histopathologic evaluation identified as an undifferentiated malignant tumor consistent with liposarcoma, without the subtype being further classified [49]. Therefore, no general conclusions regarding survival and metastatic behavior can be drawn. SW-872 has been reported to be sensitive to various chemotherapeutic agents, such as doxorubicin, cisplatin, and vinblastine, making it a representative cell line for chemosensitive STS in this study [50].

The biphasic synovial sarcoma is an aggressive STS, composed of spindle-shaped cells and epithelial-like cells, and defined by the characteristic chromosomal translocation t(X; 18) [51,52]. It most frequently appears in young adults and the prognosis, especially for metastatic disease, is poor [5,51]. RT in addition to surgery leads to an improved outcome [11]. SW-982 is a biphasic synovial sarcoma cell line [53]. Although SW-982 is generally described as chemosensitive to doxorubicin in a previous study, it demonstrated chemoresistance to doxorubicin, epirubicin, and cisplatin in its CD133+ and aldehyde dehydrogenase 1+ subgroups [54,55,56]. Therefore, in this study it represents STS with a varying sensitivity to CT.

Rhabdomyosarcoma is the most common STS in children with 70% of cases occurring in patients under the age of six [5,57]. In advanced stages there is no standard chemotherapeutic regimen and the five-year survival rates are approximately 61% in patients younger than 20 and only 27% in adults [5,22]. RT improves the outcome of localized rhabdomyosarcoma [58]. TE-671 is a cell line that was previously used as a medulloblastoma model, but was identified as a rhabdomyosarcoma in 1989 [59]. TE-671 shows various sensitivity to chemotherapeutic agents. In a previous study, treatment with cyclophosphamide or vincristine caused growth delay, while cisplatin had little effect [60]. TE-671 demonstrated marked radiosensitivity to single-dose irradiation, consistent with the radiosensitivity of localized rhabdomyosarcoma [58,60]. In this study it is used as a representative cell line for radiosensitive STS with varying chemosensitivity.

Overall, this study included one chemosensitive cell line, two cell lines with intermediate chemosensitivity and one chemoresistant cell line to cover the broad spectrum of STS responses to CT when investigating a potential new chemotherapeutic agent [61].

Despite early-phase studies, the therapeutic potential of LU in STS treatment has not been evaluated sufficiently yet, especially its effects as a monotherapy and its radiosensitizing potential have not been identified. Given the ongoing challenges in the treatment of metastatic STS, there is a clear need for more effective and better-tolerated therapeutic options. In this study, the effects of LU as a single agent and in combination with RT are tested on various STS cell lines to assess its direct antitumor activity and its radiosensitizing potential. The results may help identify LU as next-generation treatment option either as a single agent or in combination strategies such as radiochemotherapy.

## 2. Materials and Methods

### 2.1. Cell Lines and Cell Culture

The STS cell lines SW-684 (fibrosarcoma), SW-872 (liposarcoma), SW-982 (biphasic synovial sarcoma), and TE-671 (rhabdomyosarcoma) were all purchased from Cytion (Eppelheim, Germany). SW-684 was cultivated in Dulbecco’s Modified Eagle Medium Nutrient Mixture/F12 (Ham) (DMEM/F12; Thermo-Fisher Scientific, Waltham, MA, USA) with additional 1.5% N-(2-Hydroxyethyl)-piperazine-N’-(2-ethanesulfonic acid) (HEPES; Sigma-Aldrich, St. Louis, MO, USA). SW-982, TE-671 and SW-872 were all cultured in DMEM High Glucose (4.5 g/L) with L-Glutamine (Capricorn Scientific, Ebsdorfergrund, Germany). The cell culture medium for all cell lines was supplemented with 10% fetal bovine serum (FBS; Sigma-Aldrich Corporation, St. Louis, MO, USA) and 1% penicillin-streptomycin (Thermo-Fisher Scientific, Waltham, MA, USA) unless specified otherwise. The medium of SW-872 was additionally supplemented with 0.1% Sodium Pyruvate (100 mM) (Thermo-Fisher Scientific, Waltham, MA, USA). All cell lines were incubated at 37 °C and 5% CO_2_ and were passaged at regular intervals of 3–4 days to a maximum of 10 passages. Cell numbers were quantified using a CASY cell counter (Omni Life Science GmbH + Co. KG, Bremen, Germany).

### 2.2. Treatment with Lurbinectedin and Radiotherapy

LU (Selleck Chemicals GmbH, Houston, TX, USA) was diluted in dimethyl sulfoxide (DMSO; Carl Roth GmbH + Co. KG, Karlsruhe, Germany) creating a stock solution 1 with a concentration of 1000 µM. Stock solution 1 was diluted 1:10,000 in phosphate-buffered saline (PBS; Medicago AB, Uppsala, Sweden) to produce stock solution 2 (0.1 µM), which was used in the experiments. They were all stored at −80 °C in aliquots and the required amount of stock solution 2 was thawed right before using, whereas stock solution 1 was only thawed to create a new stock solution 2 when needed. A concentration of 0.5 nM LU was used for the treatment, as determined in a dose-escalation experiment beforehand (Appendix A). With this concentration a measurable effect on apoptosis/necrosis and cell cycle distribution was induced, while maintaining enough cells for the measurement in our experimen4-day tal setup. The selected concentration was consistent with previous investigations determining the synergistic effects of LU in combination with other chemotherapeutic agents in different cancer types in vitro [62,63]. LU stock solution 2 was added directly to the medium.

RT was carried out using an ISOVOLT Titan X-Ray generator (GE, Ahrensburg, Germany); cells were irradiated with 2 Gy, 3 × 2 Gy, 3 Gy, or 2 × 3 Gy depending on the experimental conditions.

Cells were treated with either LU, ionizing radiation (IR), or a combination of both. For combined treatment, IR was performed for the first time 3 h or 24 h after adding LU depending on the experimental setup. To consider solvent effects, the control was treated with DMSO (diluted 1:10,000 in PBS) matching the DMSO concentration of the LU-treated group.

### 2.3. Assay for Apoptosis/Necrosis and Cell Cycle Distribution Following Single-Dose Ionizing Radiation (4-Day Protocol)

**Figure 1:**
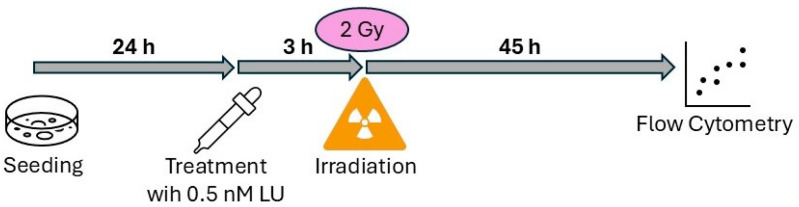
Schematic representation of the experimental setup for the assay for apoptosis/necrosis and cell cycle distribution following single-dose IR (4-day protocol).

At time point t = 0 h, 200,000 cells were seeded into T25 flasks and incubated for 24 h. At t = 24 h, the culture medium was replaced with a medium containing a reduced FBS concentration of 2% (2% FBS medium). The cells were treated with LU and/or 2 Gy IR 3 h afterwards and incubated again for 45 h (Figure 1). At t = 72 h, cells were harvested, the supernatant and the cells were collected in 15 mL falcon tubes, and centrifuged (5 min, 20 °C, 300× *g*). After deposing the supernatant, the cells were resuspended and the pellet was separated in two equal parts; one was used for apoptosis/necrosis analysis, the other half was used for cell cycle analysis. For cell cycle analysis 1 mL 2% FBS medium and 10 mL 4 °C cold ethanol (Otto Fischar GmbH + Co. KG, Saarbrücken, Germany) were added to the cells and they were stored in a dark environment at 4 °C for at least 24 h and a maximum of 10 days.

In contrast, the apoptosis/necrosis measurement was done immediately after harvesting. For that reason, the cells were resuspended in 200 µL cold Ringer’s solution (Fresenius Kabi AG, Bad Homburg, Germany). 10 µL of a master mix containing Alexa Flour 555 Annexin-V-conjugate (Annexin, A35108, Thermo-Fisher Scientific, Waltham, MA, USA) and 7-amino-actinomycin D (7AAD, 559925, BD Biosciences, Franklin Lakes, NJ, USA) in a 1:1 ratio were also pipetted to the cells. The cell suspension was mixed thoroughly by vortexing and incubated in the dark for 30 min at 4 °C.

The falcon tubes with the cell suspension for cell cycle analysis were centrifugated (5 min, 20 °C, 300× *g*) and the cells were resuspended in a master mix consisting of 1 mL cold Ringer’s solution and 3 µL Hoechst 33342 (10 mg/mL H3570, Thermo-Fisher Scientific, Waltham, MA, USA) after the supernatant was discarded. Afterwards, the solution was incubated in the dark for 1 h at 4 °C.

The cell suspension for the apoptosis/necrosis measurement as well as the cell suspension for cell cycle analysis were centrifugated (5 min, 20 °C, 300× *g*) after incubation, the supernatant was discarded, and the cells were diluted in 150 µL cold Ringer’s solution. The samples were transferred to a 96-well-plate and analyzed using the CytoFLEX S flow cytometer (flow cytometer; Beckmann Coulter, Brea, CA, USA).

The results were interpreted using Kaluza Analysis software (Beckmann Coulter, Brea, CA, USA). For the apoptosis/necrosis analysis Annexin+/7AAD+ cells were defined as necrotic, Annexin+/7AAD- cells as apoptotic and Annexin-/7AAD- as living [64]. The evaluation of the cell cycle was performed based on the nuclear DNA content of the cells [65].

### 2.4. Scratch Assay

To evaluate the effect of 0.5 nM LU, 2 Gy, and the combination of both on cellular migration, we used scratch assays [66]. Therefore, we created a clear gap in the cell layer using two-well culture-inserts (ibidiGmbH, Gräfelfing, Germany) and images of the closing gap were recorded with the life-cell-imaging system zenCell owl (innoMe GmbH, Espelkamp, Germany). One two-well silicone insert was centred in each well of a 24-well cell culture plate and 50,000 cells were seeded into each chamber. When complete confluence was reached after 30 h of incubation, the inserts were removed. The middle lining of the insert left a clear scratch between both cell layers. After fresh cultivation medium and 0.5 nM LU were added, the culture plate was incubated for three more hours, which was then followed by irradiation with 2 Gy IR (see Section 2.3). Afterwards, the culture plate was positioned ensuring the gap was visible in the field of view of the zenCell owl microscope, which automatically took images of the gap every hour. The system was stopped when the gaps in all conditions were fully closed.

The evaluation of each experiment was based on the time it took the gap of the DMSO control to close completely ($A_{control\;}=O\;{\mathrm{mm}}^{2}$). All other conditions (0.5 nM LU, 2 Gy, 0.5 nM LU + 2 Gy) were analyzed at the same timepoint to calculate their remaining scratch area ($A_{condition}$) using Biomas Software (V3.0 7/2012, Erlangen, Germany). The initial gap after the inserts were removed was 0.6 ${\mathrm{mm}}^{2}$ ($A_{initial}$). We calculated and plotted the proportion of the remaining scratch to the initial scratch.

### 2.5. Assay for Clonogenic Survival

For the assessment of clonogenic survival, cells were seeded in Petri dishes with a diameter of 6 cm at t = 0 h. Cell numbers were adjusted depending on the cell line and the treatment condition. For each treatment, the mean of two technical replicates was used. After a 24 h incubation period, LU was added to the respective cells and 3 h later the cells designated for IR received a dose of 2 Gy. At t = 72 h, the cultivation medium was exchanged to terminate the exposure of the cells to LU. The cells were incubated again for 10 to 14 days until colonies of at least 50 cells were formed in all Petri dishes. Using methylene blue (Carl Roth GmbH + Co. KG, Karlsruhe, Germany) the colonies were stained for 30 min at room temperature after the medium was discarded. After removing the methylene blue, the Petri dishes were washed using distilled water and left to dry for 24 h. To determine if the number of cells able to proliferate indefinitely changed with receiving a treatment, all colonies consisting of more than 50 cells were defined as descending from a clonogenic cell and counted manually [67].

### 2.6. Assay for Apoptosis/Necrosis and Cell Cycle Distribution Combined with Clonogenic Survival Following Fractionated Ionizing Radiation (6-Day Protocol)

**Figure 2:**
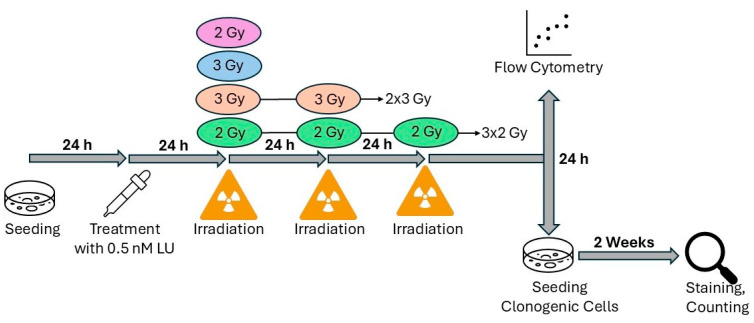
Schematic representation of the experimental setup for the assay for apoptosis/necrosis and cell cycle distribution combined with clonogenic survival following fractionated IR (6-day protocol).

In this experimental setup, the duration between LU treatment and IR was prolonged and the IR conditions of the cells were expanded. Cells received either a single dose of 2 Gy or 3 Gy, or a fractionated IR scheme of 3 × 2 Gy or 2 × 3 Gy with a 24 h interval between each fraction. 200,000 cells were seeded into T25 flasks and incubated for 24 h. After exchanging the medium to 2% FBS medium at t = 24 h, LU was added to the LU-receiving cells, DMSO was added to the control, then they were incubated for another 24 h. At t = 48 h, all cells designated for IR treatment received their first dose, at t = 72 h the cells undergoing fractionated IR were exposed to their second dose, and at t = 96 h a final fractionated IR for the cells assigned to be irradiated three times was performed. In between those treatments and for 24 h after the last treatment the cells were incubated (Figure 2).

After collecting the cells and the supernatant in 15 mL falcon tubes, 3 mL of each suspension were separated for clonogenic survival. The remaining cell suspension was prepared and analyzed using the flow cytometer in the same manner as described in detail in the assay for apoptosis/necrosis and cell cycle measurement following single-dose IR (Section 2.3).

While measurements were made using the flow cytometer, the cell suspension set aside for analyzing the clonogenic survival was centrifugated (5 min, 20 °C, 300× *g*) and resuspended in 5 mL nutrient medium after discarding the supernatant. A cell number adjusted to the cell line and treatment condition was seeded into Petri dishes (diameter = 6 cm) and incubated for 10 to 14 days until colonies containing at least 50 cells had formed in all treatment groups. Following the same procedure as described in the assay for clonogenic survival (Section 2.5) the colonies were stained and counted.

### 2.7. Statistics

All experiments were performed independently at least four times. Statistical significance was determined using the two-tailed Mann–Whitney U test with GraphPad Prism 9.5.1 (GraphPad Software, San Diego, CA, USA), which was also used to generate the graphs. *p*-values < 0.05 were considered significant. Comparisons were made between 0.5 nM LU/0.5 nM LU + IR and the control to assess the effect of LU, and between 0.5 nM LU + IR and 0.5 nM LU to determine the additional effect of the combination.

## 3. Results

### 3.1. Apoptosis/Necrosis Induction by Lurbinectedin and Lurbinectedin + 2Gy Following the 4-Day Protocol

**Figure 3:**
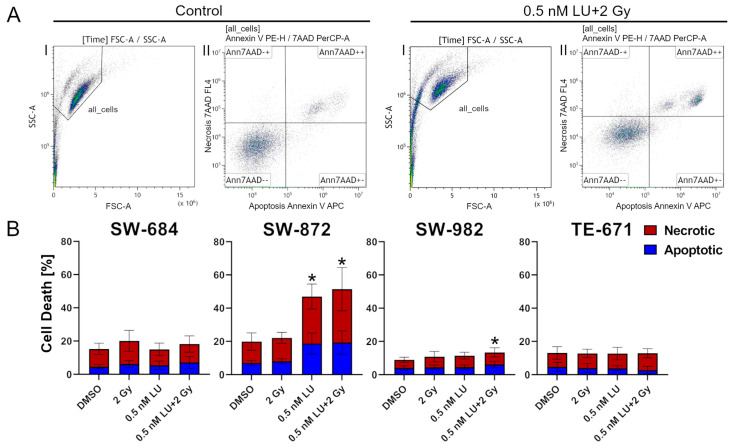
Flow cytometry analysis of apoptosis/necrosis following the 4-day protocol. The sum of apoptosis and necrosis indicates the effect of each treatment. (**A**) Representative gating strategy applied on SW-982 in the control and 0.5 nM LU + 2 Gy. First, cell debris was excluded. Then, the percentage of apoptotic cells (Ann7AAD+−) and necrotic cells (Ann7AAD++) was determined. (**B**) The graphs display the percentage of apoptotic and necrotic cells after treatment for each cell line. The cells have been treated with 2 Gy, 0.5 nM LU, 0.5 nM LU + 2 Gy or the same volume DMSO (control) as used for 0.5 nM LU. The mean has been calculated from four independent experiments. The standard deviation is displayed by error bars. The two-tailored Mann–Whitney U test was used to determine the significance between the control and 0.5 nM LU; the control and 0.5 nM LU + 2 Gy; 0.5 nM LU and 0.5 nM LU + 2 Gy. * indicates a significant value *p* = 0.029 compared to the control. There were no significant differences between 0.5 nM LU and 0.5 nM LU + 2 Gy. Abbreviations: LU, lurbinectedin; Ann, Annexin; 7AAD, 7-amino-actinomycin D.

The 4-day protocol was used initially to determine the effect of 0.5 nM LU or the combination of 0.5 nM LU with 2 Gy in general. Apoptosis/necrosis was determined with flow cytometry using Annexin V and 7AAD to differentiate between apoptosis and necrosis. At first, cell debris was excluded. Afterwards, living cells were defined as double negative (Ann7AAD−−), apoptotic cells as Annexin V positive (Ann7AAD+−), and necrotic cells as double positive (Ann7AAD++) (Figure 3A). To compare apoptosis/necrosis resulting from the different treatments the sum of apoptosis and necrosis was considered. The difference between the control compared to 0.5 nM LU and 0.5 nM LU + 2 Gy was cell line specific. In SW-684 and TE-671, there was no significant increase in apoptosis/necrosis after any of the treatments. In contrast, 0.5 nM LU and 0.5 nM LU + 2 Gy increased the apoptosis/necrosis of SW-872 significantly compared to the control (*p* = 0.029). While 0.5 nM LU did not increase the apoptosis/necrosis of SW-982 significantly (*p* = 0.200), the combination of 0.5 nM LU + 2 Gy had a significant effect (*p* = 0.029). 0.5 nM LU + 2 Gy did not have a clear additional effect compared to 0.5 nM LU as a monotherapy in all cell lines (Figure 3B).

### 3.2. Effects of Lurbinectedin and Lurbinectedin + 2Gy on the Cell Cycle Following the 4-Day Protocol

**Figure 4:**
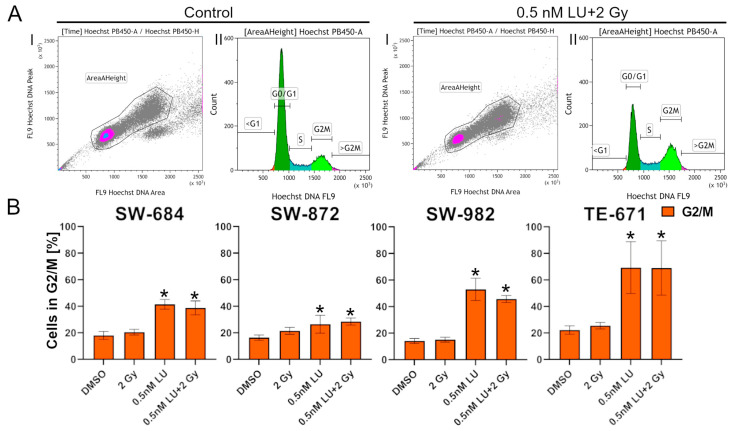
Flow cytometry analysis of cell cycle distribution following the 4-day protocol. The fraction of cells in the G2/M phase indicates the effect of each treatment. (**A**) Representative gating strategy for Hoechst staining applied on SW-684 in the control and 0.5 nM LU + 2 Gy. First, cell debris and doublets were excluded. Then, the percentage of cells in the G2/M phase was determined based on their DNA content. (**B**) The graphs display the percentage of cells in the G2/M phase after treatment for each cell line. The cells have been treated with 2 Gy, 0.5 nM LU, 0.5 nM LU + 2 Gy or the same volume DMSO (control) as used for 0.5 nM LU. The mean has been calculated from four independent experiments. The standard deviation is displayed by error bars. The two-tailored Mann–Whitney U test was used to determine the significance between the control and 0.5 nM LU; the control and 0.5 nM LU + 2 Gy; 0.5 nM LU and 0.5 nM LU + 2 Gy. * indicates a significant value *p* = 0.029 compared to the control. There were no significant differences between 0.5 nM LU and 0.5 nM LU + 2 Gy. Abbreviations: LU, lurbinectedin.

This experiment was used to assess if 0.5 nM LU or 0.5 nM LU + 2 Gy affect the cell cycle and especially the radiosensitive G2/M phase. The cell cycle distribution was determined with flow cytometry using Hoechst staining. The cell debris and doublets were excluded, and the gating was performed based on the nuclear DNA content of the cells (Figure 4A). The significance of the treatments was determined by comparing the fraction of cells in the G2/M phase. All cell lines showed a significant increase in the G2/M phase after treatment with 0.5 nM LU or 0.5 nM LU + 2 Gy (*p* = 0.029) compared to the control. 0.5 nM LU + 2 Gy did not have a significant additional effect compared to 0.5 nM LU as a monotherapy. In contrast, a slight decrease in cells in the G2/M phase could be seen in all cell lines except SW-872 when irradiating additionally after the treatment with 0.5 nM LU (Figure 4B).

### 3.3. Scratch Assay

**Figure 5:**
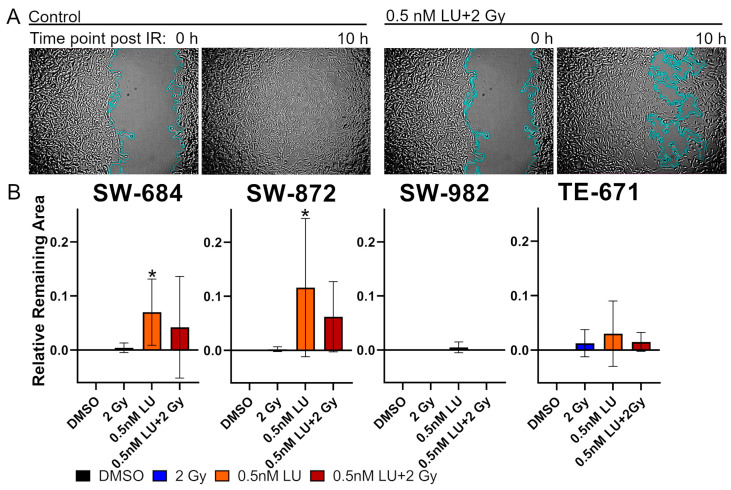
Scratch assay analysis. (**A**) Representative images of the control and 0.5 nM Lu + 2 Gy treatment group after IR and after the gap in the control was fully closed using SW-684. (**B**) The graphs display the relative remaining area after the gap has been fully closed in one treatment scheme. The cells have been treated with 2 Gy, 0.5 nM LU, 0.5 nM LU + 2 Gy or the same volume DMSO (control) as used for 0.5 nM LU. The mean has been calculated from five independent experiments for SW-684 and SW-872 and four independent experiments for SW-982 and TE-671. The standard deviation is displayed by error bars. The two-tailored Mann–Whitney U test was used to determine the significance between the control and 0.5 nM LU; the control and 0.5 nM LU + 2 Gy; 0.5 nM LU and 0.5 nM LU + 2 Gy. * indicates a significant value *p* = 0.048 compared to the control. There were no significant differences between 0.5 nM LU and 0.5 nM LU + 2 Gy. Abbreviations: LU, lurbinectedin; IR, ionizing radiation.

To determine if 0.5 nM LU has an effect on cell migration, the scratch assay was performed and the cell migration was monitored using a life-cell-imaging system (Supplementary Video S1). The relative scratch area was measured as soon as the cells in the control had fully closed the area and was normalized to the area in the beginning ($\frac{remaining\;area}{area\;in\;the\;beginning}$) (Figure 5A).

In SW-684 and SW-872 cell migration was suppressed by 0.5 nM LU compared to the control (*p* = 0.048), while it did not affect the cell migration in SW-982 and TE-671 compared to the control (*p* > 0.999).

0.5 nM LU + 2 Gy did not have a significant effect on all cell lines compared to the control and compared to 0.5 nM LU as a monotherapy (Figure 5B).

### 3.4. G2/M Cell Cycle Distribution

To exploit the radiosensitizing effect of LU causing a G2/M arrest, we determined when G2/M arrest is the most pronounced after adding 0.5 nM LU. Two of the four cell lines were selected exemplary for analysis. Cells were measured with flow cytometry using Hoechst staining 3 h, 12 h, 24 h, 48 h, or 72 h after treatment with 0.5 nM LU or the control. The same gating strategy as described in the 4-day protocol (Section 3.2) was used.

**Figure 6:**
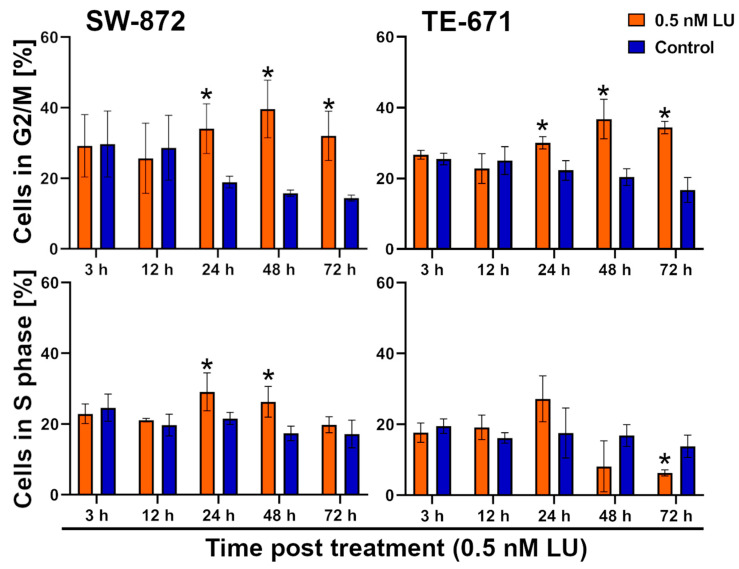
Flow cytometry analysis of the G2/M phase and S phase at different time points. The fraction of cells in the G2/M phase and S phase indicates the effect of each treatment. The percentage of cells in the G2/M phase and S phase was determined based on their DNA content. The graphs display the percentage of cells in the G2/M phase (I) and S phase (II) after treatment for each cell line. The cells have been treated with 0.5 nM LU or the same volume DMSO (control) as used for 0.5 nM LU and measured 3 h, 12 h, 24 h, 48 h, or 72 h after LU-treatment. The mean has been calculated from four independent experiments. The standard deviation is displayed by error bars. The two-tailored Mann–Whitney U test was used to determine the significance between 0.5 nM LU and the control for each time point. * indicates a significant value *p* = 0.029 compared to the control. Abbreviations: LU.

After three and twelve hours, a similar percentage of cells were in the G2/M phase in both control group and the group treated with 0.5 nM LU. At 24 h, LU led to an increased G2/M arrest in the group treated with 0.5 nM LU. This effect was even more pronounced after 48 h. At 72 h, the G2/M arrest began to decline, but an accumulation of cells in G2/M phase was still evident. The period between 24 h and 72 h post LU-treatment appears to be the best for irradiating during the LU- induced G2/M arrest in both cell lines (*p* = 0.029). In the control, the percentage of cells in the G2/M phase kept decreasing over time (Figure 6).

**Figure 7:**
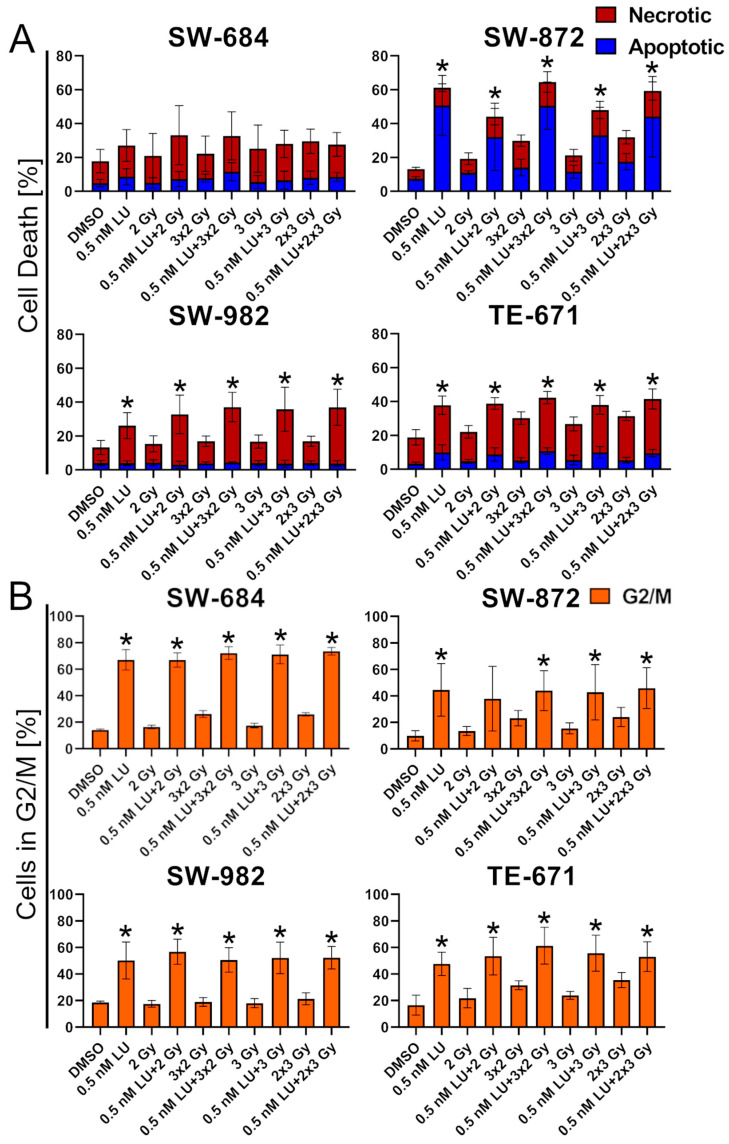
Flow cytometry analysis of necrosis and apoptosis and the cell cycle distribution following the 6-day protocol. The sum of apoptosis and necrosis indicates the effect of each treatment on apoptosis/necrosis. The fraction of cells in the G2/M phase indicates the effect of each treatment on the cell cycle. The graphs display the percentage of (**A**) apoptotic and necrotic cells and (**B**) of cells in the G2/M phase after the treatment for each cell line. The cells have been treated with IR (2 Gy; 3 × 2 Gy; 3 Gy; 2 × 3 Gy), 0.5 nM LU, 0.5 nM LU + IR (2 Gy; 3 × 2 Gy; 3 Gy; 2 × 3 Gy) or the same volume DMSO (control) as used for 0.5 nM LU. The mean has been calculated from four independent experiments. The standard deviation is displayed by error bars. The two-tailored Mann–Whitney U test was used to determine the significance between the control and 0.5 nM LU; the control and 0.5 nM LU + IR; 0.5 nM LU and 0.5 nM LU + IR. * symbolizes a significant value *p* = 0.029 compared to the control. There were no significant differences between 0.5 nM LU and 0.5 nM LU + IR. Abbreviations: LU, lurbinectedin.

To increase the induction of apoptosis/necrosis, this knowledge was used to irradiate the cells when they are in the radiosensitive G2/M-phase (6-day protocol); however, even with the optimized treatment scheme, the induction of apoptosis/necrosis was still limited and in SW-872 single-dose IR even seemed to interfere negatively with the effect of lurbinectedin on apoptosis/necrosis (Figure 7). Therefore, the S-Phase was analyzed retrospectively to evaluate how the radiosensitizing effect of the G2/M arrest was bypassed. In SW-872, there was a significant S phase cell cycle arrest 24 h and 48 h after LU treatment compared to the control (*p* = 0.029). 72 h post-treatment this effect was already diminished again. In TE-671, an increase in cells in the S phase by LU was observed at 24 h compared to the control. Notably, at 48 h and 72 h, the treatment had the opposite effect and a lower percentage of cells was in the S phase in the LU group compared to the control. This reduction was significant at 72 h post treatment (*p* = 0.029) (Figure 6).

### 3.5. Effects of Lurbinectedin and the Combination of Lurbinectedin and Fractionated Ionizing Radiation on Apoptosis/Necrosis and Cell Cycle Arrest

Since LU triggers G2/M arrest, IR was administered accordingly in a fractionated regimen with 3 × 2 Gy and 2 × 3 Gy in order to treat the cells in their most radiosensitive state. Compared to the experimental setup in Figure 3B, in this experiment the incubation time after LU-treatment was prolonged to 96 h instead of 48 h, and the first irradiation was performed 24 h after LU-treatment instead of 3 h. In SW-684, apoptosis/necrosis was low after LU-treatment with 16.84% in the control and 28.01% after treatment with 0.5 nM LU. No relevant additive effect was observed when 0.5 nM LU + IR was compared to 0.5 nM LU as a monotherapy.

In all other cell lines, 0.5 nM LU and all combinations of 0.5 nM LU and IR led to a significant increase in apoptosis/necrosis compared to the control (*p* =0.029).

No additive effect on apoptosis/necrosis was observed in SW-872 when comparing 0.5 nM LU as a monotherapy and 0.5 nM LU + IR. In contrast, 0.5 nM LU + 2 Gy and 0.5 nM LU + 3 Gy resulted in less apoptosis/necrosis than 0.5 nM LU alone.

Although the difference between 0.5 nM LU and 0.5 nM LU + IR was not statistically significant in SW-982, the combination of 0.5 nM LU + IR showed an additive effect on apoptosis/necrosis (26.2 % after 0.5 nM LU compared to 36.9% after 0.5 nM LU + 2 × 3 Gy). In TE-671, there was no additive effect between 0.5 nM LU as a monotherapy and 0.5 nM LU + IR (Figure 7A).

The G2/M phase increased significantly in all cell lines at 0.5 nM LU and 0.5 nM LU + IR compared to the control (*p* = 0.029), except for the 0.5 nM LU + 2 Gy treatment in the SW-872 cell line (*p* = 0.057). In SW-684 and SW-982, there was no additive effect between 0.5 nM LU and 0.5 nM LU + IR. In SW-872, 0.5 nM LU + 2 Gy and 0.5 nM LU + 3 Gy resulted in a reduced percentage of cells being in the G2/M phase compared to 0.5 nM LU alone.

In TE-671, there was a slight additive effect (44.7% after 0.5 nM LU compared to 61.0% after 0.5 nM LU + 3 × 2 Gy) (Figure 7B).

### 3.6. Effects of Lurbinectedin and the Combination of Lurbinectedin with Ionizing Radiation on the Clonogenic Survival

**Figure 8:**
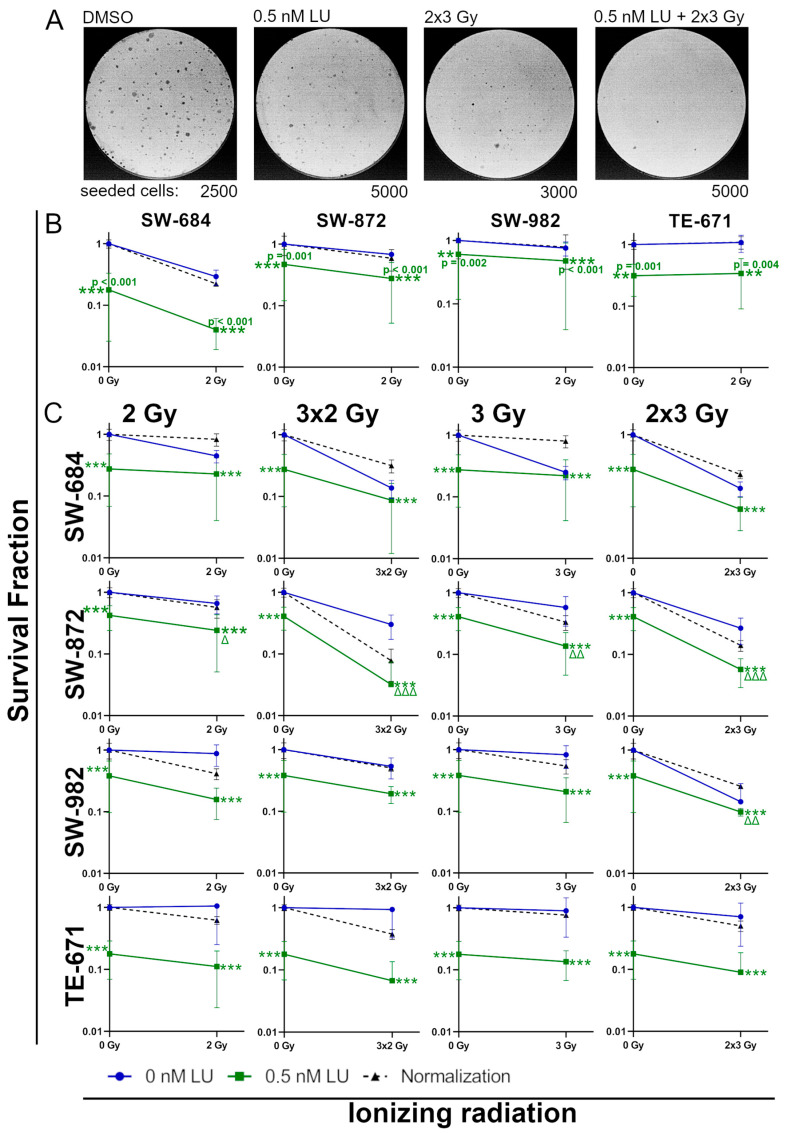
Clonogenic survival. (**A**) Representative images of the clonogenic survival assay with stained Petri dishes after 11 days of incubation using SW-872. (**B**) Survival fraction as estimated by the clonogenic survival assay. The cells have been treated with 2 Gy, 0.5 nM LU, 0.5 nM LU + 2 Gy or the same volume DMSO (control) as used for 0.5 nM LU. 0.5 nM LU data were normalized to the control (dotted black line). IR was applied 3 h after LU-treatment (corresponding to the 4-day protocol). **; *** indicate a significant value compared to the control. There were no significant differences between 0.5 nM LU and 0.5 nM LU + 2 Gy. (**C**) The graphs display the surviving fraction after treatment following the 6-day protocol. The cells have been treated with 0.5 nM LU, 0.5 nM LU + IR (2 Gy; 3 × 2 Gy; 3 Gy; 2 × 3 Gy) or the same volume DMSO (control) as used for 0.5 nM LU. 0.5 nM LU data was normalized to the control. IR was applied in 24 h intervals after LU-treatment. The mean has been calculated from four independent experiments. The standard deviation is displayed by error bars. The two-tailored Mann–Whitney U test was used to determine the significance between the control and 0.5 nM LU; the control and 0.5 nM LU + IR; 0.5 nM LU and 0.5 nM LU + IR (applicable for **B** and **C**). *** indicates a significant value *p* < 0.001 compared to the control. Δ indicates a significant value *p* = 0.048; ΔΔ indicates a significant value *p* = 0.003 in SW-872 and *p* = 0.007 in SW-982; ΔΔΔ indicates a significant value *p* < 0.001 compared to 0.5 nM LU. Abbreviations: LU, lurbinectedin.

The assay for clonogenic survival was used to determine the survival fraction of cells able to proliferate indefinitely after being treated with 0.5 nM LU or 0.5 nM LU + IR (2 Gy; 3 × 2 Gy; 3 Gy; 2 × 3 Gy) (Figure 8A). To get a deeper insight into the efficacy of our applied treatment scheme, we used the same experimental setup for clonogenic cell assay as for flow cytometry. This allows conclusions about cell viability beyond induction of apoptosis/necrosis.

To determine if the combination of 0.5 nM LU + IR has a radiosensitizing effect, the LU-treated cells were normalized to the DMSO control by shifting the 0.5 nM LU line to the same starting point as the cells treated with DMSO, making it easier to compare the additional effect of the IR treatment.

In the first experiment, the cells receiving RT were irradiated 3 h after treatment with 0.5 nM LU (corresponding to the 4-day protocol).

0.5 nM LU (SW-684 *p* < 0.001; SW-872 *p* = 0.001; SW-982 *p* = 0.002; TE-671 *p* = 0.001) and 0.5 nM LU + 2 Gy (SW-684 *p* < 0.001; SW-872 *p* < 0.001; SW-982 *p* < 0.001; TE-671 *p* = 0.004) clearly decreased survival in all cell lines compared to the control. There were no significant differences between 0.5 nM LU as a monotherapy and 0.5 nM LU + 2 Gy, but in SW-684, SW-872, and SW-982, adding 2 Gy to 0.5 nM LU had an additional effect on the decrease in clonogenic cells. In SW-684 and SW-872, an additional radiosensitizing effect could be seen when combining 0.5 nM LU with 2 Gy. In SW-982 and TE-671, 0.5 nM LU did not have a radiosensitizing effect. Among all cell lines, the survival fraction decreased the least after IR in TE-671 (Figure 8B).

In the second experiment, fractionated treatment was studied using the 6-day protocol. All cell lines had a significant decrease in survival fraction after being treated with 0.5 nM LU (*p* < 0.001) and 0.5 nM LU + IR (*p* < 0.001) compared to the control.

In SW-684, LU-treatment reduced the sensitivity of the cells to IR. Still, an additional decrease in clonogenicity could be seen when adding fractionated IR (3 × 2 Gy, 2 × 3 Gy) after LU-treatment compared to 0.5 nM LU alone, although not significant.

In SW-872, 0.5 nM LU + IR led to a significant additional decrease in survival fraction in all IR schemes (0.5 nM LU + 2 Gy *p* = 0.048; 0.5 nM LU + 3 × 2 Gy *p* < 0.001; 0.5 nM LU + 3 Gy *p* = 0.003; 0.5 nM LU + 2 × 3 Gy *p* < 0.001) compared to 0.5 nM LU as a monotherapy. Furthermore, the sensitivity of the cells to IR increased notably after LU-treatment in 0.5 nM LU + 3 × 2 Gy, 0.5 nM LU + 3 Gy, and 0.5 nM LU + 2 × 3 Gy.

In SW-982, 0.5 nM LU + IR resulted in a significant additional decrease in clonogenic cells in the 0.5 nM LU + 2 × 3 Gy treatment (*p* = 0.007) compared to 0.5 nM LU as a monotherapy. In the other treatment schemes, an additional decrease in clonogenic cells was also seen when adding IR to the treatment with 0.5 nM LU, although it was not significant.

In TE-671, 0.5 nM LU + IR caused no significant decrease in survival fraction compared to 0.5 nM LU as a monotherapy, but an additional effect could be seen in all treatment schemes, especially with fractionated IR. In all IR schemes, LU had a radiosensitizing effect on TE-671 (Figure 8C).

## 4. Discussion

### 4.1. The Effects of LU as a Monotherapy

**Figure A1:**
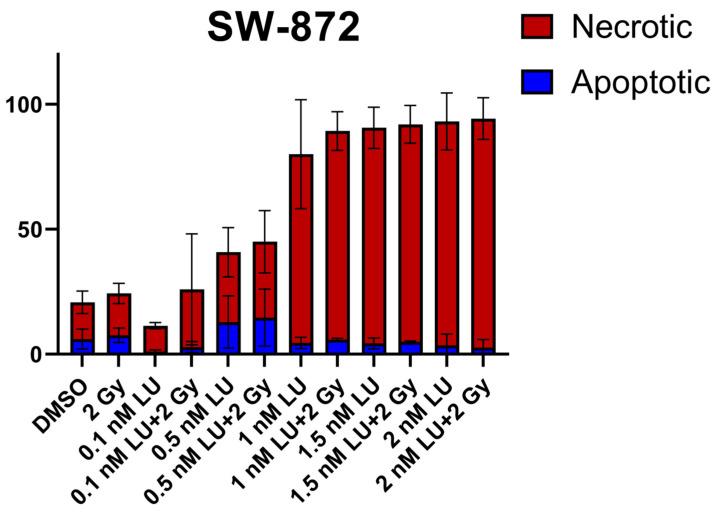
Flow cytometry analysis of apoptosis/necrosis following the 4-day protocol on SW-872 for dose-finding. The sum of apoptosis and necrosis indicates the effect of each treatment. The graph displays the percentage of apoptotic and necrotic cells after treatment. The cells have been treated with 2 Gy, 0.1 nM LU, 0.5 nM LU, 1 nM LU, 1.5 nM LU, 2 nM LU, 0.1 nM LU + 2 Gy, 0.5 nM LU + 2 Gy, 1 nM LU + 2 Gy, 1.5 nM LU + 2 Gy, 2 nM LU + 2 Gy or the same volume DMSO (control) as used for 2 nM LU. The mean has been calculated from two independent experiments except the treatment with 0.5 nM LU and 0.5 nM LU + 2 Gy and the respective control group, which were repeated four independent times. The standard deviation is displayed by error bars. Abbreviations: LU, lurbinectedin.

An important result of this study was that LU triggered a marked increase in G2/M arrest in all four cell lines. G2/M arrest is normally followed by cell apoptosis [68] (Figure 4B). This G2/M arrest indicates that LU had a cytotoxic effect on the STS cell lines [69]. However, apoptosis/necrosis did not increase with the combined treatment in most cell lines (Figure 3B), although there was a significant reduction in clonogenic cells (Figure 8B). For agents interfering with the cell cycle, like LU, cytotoxicity is known to be dependent not only on the doubling time, but also exposure time to CT to ensure that enough cells enter the sensitive phase during treatment [70,71]. LU is normally administered at a standard dose of 3.2 mg/m^2^, for example for treatment of SCLC [40]. Our used concentration was defined specifically for our experiments. The 0.5 nM LU concentration used in our in vitro experiments represents a low dose suitable for testing the cellular response under controlled experimental conditions in sarcoma cells (Figure A1), rather than being directly transferrable to the in vivo dose used in the clinics. LU has a reported terminal half-life of 46.59 h [33,40,72]. Given that 94% to 97% of a drug is eliminated from the body after approximately 4 to 5 half-lives, LU remains in systemic circulation for around 186.36 h to 232.95 h [73]. This long systemic exposure supports the hypothesis that the cytotoxic effects of LU require a longer duration to manifest completely. This suggests that the exposure duration of 48 h in the 4-day protocol may not be long enough to fully capture the impact of LU on the apoptosis/necrosis, even though it already showed an effect on the clonogenic survival. Furthermore, apoptosis typically following the G2/M phase requires additional time to manifest [74]. It is therefore possible that the 4-day protocol only captured the initial cell cycle arrest, while the subsequent induction of apoptosis had not yet occurred in enough cells.

In the scratch assay, which was used to assess whether LU inhibits cell migration and therefore reduces metastatic formation, the results were inconsistent. While a reduction in migration was observed in two cell lines, the other cells treated with LU migrated similarly fast as in the control, indicating that LU does not have a consistent notable impact on migratory behavior. Combining LU with IR did not increase the delay in cell migration compared to the monotherapy (Figure 5B).

In the 6-day protocol, with a prolonged exposure duration to LU of 96 h, a more pronounced effect was observed in apoptosis/necrosis, while the effect on the cell cycle remained strong. Specifically, apoptosis/necrosis increased significantly in all cell lines except SW-684 after 0.5 nM LU, where an increase was also observed although it was not statistically significant. This protocol was therefore more suitable for measuring the effect of 0.5 nM LU on apoptosis/necrosis and confirmed its strong cytotoxic activity, including G2/M phase arrest and induction of apoptosis/necrosis. Only in SW-684, the effect of LU on apoptosis/necrosis was not satisfactory, which may be related to the chemoresistant tendencies this cell line already demonstrated in previous in vitro studies [45,46]. For future experiments, it would be important to further extend the duration of treatment with LU in order to reflect the in vivo exposure even better and to investigate whether apoptosis/necrosis continues to increase over time.

Also, 0.5 nM LU had a strong impact on the survival of clonogenic cells and caused a significant decrease in survival fraction in all cell lines meaning that less cells were able to proliferate indefinitely due to the treatment [67].

LU demonstrated a notable impact on the selected STS cell lines by inducing G2/M arrest, increasing apoptosis/necrosis, and reducing the number of clonogenic cells. These findings confirm its potential as a new chemotherapeutic agent for the treatment of STS. However, SW-684 did not react as strongly as the other cell lines to the treatment; increasing the concentration of LU could result in a more pronounced effect in this particularly chemoresistant cell line [45,46]. Furthermore, LU does not prevent cell migration in two of the cell lines, suggesting it may lack consistent inhibitory effects on metastatic progression. Future studies could investigate whether other STS subtypes have a similar resistance to LU as observed in SW-684. Additionally, evaluating the interaction of LU with established chemotherapeutic agents such as doxorubicin could help identify potential synergistic effects in combination therapies and broaden the therapeutic options for STS patients even further [75]. In addition, in vivo studies are needed to confirm the effects of LU on STS in a clinical setting.

### 4.2. The Effects of LU in Combination with RT

In the 4-day protocol a G2/M arrest was observed in the cell cycle, a phase known for its heightened radiosensitivity [76]. Although the G2/M arrest could be seen in the cell cycle measurement, there was no significant increase in apoptosis/necrosis and G2/M arrest when irradiating with 2 Gy 3 h after adding 0.5 nM LU (Figure 3B and Figure 4B). In the assay for clonogenic survival, an additional decrease in clonogenic cells could be achieved when adding IR to the LU-treated cells with the exception of TE-671, which did not show any response to IR at all, suggesting that this cell line may be strongly resistant to RT (Figure 8B).

Given the strong G2/M block caused by LU, we anticipated a significant increase in apoptosis/necrosis when adding IR to the treatment with 0.5 nM LU. In the 4-day protocol, no increase in apoptosis/necrosis was observed in the combination therapy compared to LU as a monotherapy. Therefore, we aimed to further optimize the treatment protocol.

Monitoring the G2/M arrest induced by LU over time made it evident that the IR had initially been performed at a suboptimal time point (Figure 6). After adjusting the protocol accordingly and irradiating 24 h after LU-treatment for the first time, and in the fractionated IR also 48 h and/or 72 h after LU-treatment, where the G2/M block caused by LU was the highest, the combination of 0.5 nM LU + IR still did not cause the increase in apoptosis/necrosis and G2/M arrest we anticipated, compared to LU as a monotherapy (Figure 7A,B). In contrast, single-dose IR seemed to interfere negatively with the effects of LU in SW-872 and adding IR caused a decrease in apoptotic/necrotic cells compared to 0.5 nM LU alone. A possible explanation could be that IR interferes with the drug-induced G2/M arrest, a phenomenon already discovered when combining antimicrotubule agents with IR [77].

Another possible explanation was discovered after monitoring the S phase over time. The cells undergo an S phase arrest strongest 24 h after treatment. As the S phase is the least radiosensitive phase, this may counteract the radiosensitizing effect caused by the G2/M arrest [78]. In TE-671, this arrest had subsided at t = 48 h and instead of an S phase arrest, a reduction in cells in S phase was observed. In SW-872, the S phase arrest was diminished at t = 72 h. These findings suggest that irradiating at an even later timepoint might be more effective. This assumption is supported further when looking at the clonogenic cell survival data. In both cell lines, the 3 × 2 Gy IR scheme caused the most decrease in clonogenic cells of all treatment schemes, which may be explained by the last IR taking place when the S phase arrest had already worn off. In contrast, single-dose IR was not as effective and sometimes even counteracted with the effect of LU, likely due to being performed while the S phase arrest was the strongest at t = 24 h.

It should also be noted that our flow cytometry measurements of apoptosis/necrosis represent only a part of the cell death mechanisms induced by RT and mitotic catastrophe, also a major cell death induced by RT, was not measured in this assay [79].

In the scratch assay additional IR did not have a beneficial effect.

However, in the second experimental setup with the optimized timepoint for IR and a prolonged LU-treatment, the reaction of cells in the assay for clonogenic survival changed. The reaction to 0.5 nM LU + IR was still heterogenous in the different cell lines, but in all cell lines an additional reduction in survival fraction could be seen when comparing 0.5 nM LU + IR to 0.5 nM as a monotherapy. In SW-684, the effect of IR was reduced when combined with 0.5 nM LU compared to IR as a monotherapy. Still, with the exception of the 0.5 nM LU + 3 Gy treatment scheme in SW-684, among all treatment groups the treatment with 0.5 nM LU + IR consistently resulted in the strongest decrease in clonogenic cells. This experiment was the first time we had a consistent additional effect on all sarcoma cell lines when combining LU with IR compared to LU as a monotherapy. Notably, TE-671 demonstrated radiosensitivity for the first time in the clonogenic survival assays presented in this study after being treated with LU according to the second experimental setup (Figure 8C).

In conclusion, the additional effect of combining LU with IR did not have the notable impact we expected on apoptosis/necrosis and cell cycle compared to LU alone. Single-dose treatment sometimes even interfered negatively with the effects of LU in the assay measuring apoptosis/necrosis. However, combining LU with IR caused a clear decrease in the survival of clonogenic cells. Although most additional effects did not reach statistical significance when combining 0.5 nM LU + IR compared to 0.5 nM LU alone, an additive decrease in survival fraction could be clearly observed in all cell lines. This suggests that repeated or prolonged treatment with the combination of 0.5 nM LU + IR may lead to potentially therapeutically relevant outcomes in most STS cell lines. When considering the S phase arrest taking place 24 h post-treatment, starting the fractionated IR at 72 h instead of 24 h after LU-treatment could increase the effect of IR further. When comparing the different IR schemes, it becomes evident that fractionated IR is consistently more effective across all cell lines. This highlights the relevance of fractionated IR in future treatment strategies and experimental designs in combination with LU, while single-dose treatment interferes negatively with the effects of LU in some of the experiments. Since LU monotherapy is already highly effective, combining single-dose treatment with LU suggests that LU may not be suitable for use in combination with IR, especially when irradiating while the S phase arrest takes place.

### 4.3. The Heterogenic Reaction of the Different STS Cell Lines

Summarizing all results, LU induces apoptosis/necrosis, cell cycle arrest and reduced clonogenic survival in three of four cell lines. The combination therapy showed promising additional effects on clonogenicity, especially when using fractionated IR. SW-684 appears to be the least sensitive cell line to LU. LU had a significant impact on its cell cycle but did not significantly affect apoptosis/necrosis. Although the combination of 0.5 nM LU + IR caused an additional reduction in clonogenic survival in the fractionated IR scheme, there was no satisfactory reaction to single-dose IR and LU decreased the reaction of the cell line to IR. Taking into account the CT resistance, this particular cell line may require higher doses of LU and IR to cause an adequate reaction to the treatment [45,46].

In the scratch assay the cell lines reacted heterogeneously to treatment with LU, indicating that LU does not have a consistent effect on cell migration.

Given the heterogeneity of STS, it is important to note that not all STS subtypes respond equally to IR, LU or the combination of both [11]. Therefore, the findings of this study cannot be easily applied to every STS without further testing of the specific subtype. However, the fact that all cell lines responded to LU in apoptosis/necrosis, cell cycle and clonogenic survival, three of them with satisfactory sensitivity, and all of them showed an additional decrease in clonogenic survival when adding IR to 0.5 nM LU indicates a promising potential for broader application across STS types.

The availability of well characterized sarcoma cell lines limits the interpretability of the presented study. The four included cell lines represent only a fraction of over 50 histological sarcoma subtypes [2]. Moreover, the subtypes of SW-684 and SW-872 have not been further classified [44,49]; therefore, conclusions regarding the behavior of the fibrosarcoma and liposarcoma cell line in a clinical setting cannot be drawn. To further improve the knowledge about the radiosensitizing effect of LU on different sarcoma subtypes and selection of responder populations, it is necessary to investigate more cell lines from well-defined subpopulations. However, we gained first insights that LU in combination with ionizing radiation could be clinically relevant in the future for treating sarcomas.

## 5. Conclusions

LU has a strong effect on the selected STS cell lines by inducing G2/M cell cycle arrest, promoting apoptosis/necrosis, and reducing the number of clonogenic cells except for SW-684. LU has a potential influence on migration, but not on all cell lines. These findings suggest that LU may be a promising candidate as a new chemotherapeutic agent in STS treatment but may not be suitable for every type of STS and does not seem to have a consistent inhibitory effect on cell migration. However, given that three of the four tested cell lines showed a response in apoptosis/necrosis, G2/M arrest, and decrease in clonogenic survival, it is reasonable to expect that LU may be broadly effective across various STS subtypes.

Our expectation that the combination of LU + IR would have an additional effect on apoptosis/necrosis or cell cycle compared to 0.5 nM LU was not confirmed in either of our experimental setups, and single-dose IR even showed an occasional antagonistic interaction with the effects of LU. The additional effect of IR on the decrease in clonogenic cells after treatment with LU was consistent in the fractionated IR scheme, suggesting that the combination of LU with fractionated IR may have a promising effect. These findings represent the first evidence of an interaction between LU and IR that may serve as a basis for further investigations on the radiosensitizing effect of LU.

## Data Availability

All data generated and presented during this study are available from the corresponding author on reasonable request.

## Supplementary Materials

The following supporting information can be downloaded at: https://www.mdpi.com/article/10.3390/cancers17172930/s1, Video S1: The cell migration was monitored using a life-cell-imaging system.

## Abbreviations

The following abbreviations are used in this manuscript:

STS	Soft Tissue Sarcoma
LU	Lurbinectedin
IR	Ionizing radiation
RT	Radiotherapy
CT	Chemotherapy

## Appendix A

To determine the LU-concentration for our experiments, the protocol described in Section 2.3. was followed. SW-872 cells were exposed to a range of different LU-concentrations to identify the most suitable dose for further experiments. The aim was to select a concentration that did not induce excessive apoptosis/necrosis, which made it possible to observe the additional effects of irradiation or further optimization of the experimental setup. As these measurements served only as orientation, they were not repeated four independent times, except for the treatment with 0.5 nM LU, 0.5 nM LU + 2 Gy, and the respective control group, which were repeated four independent times to ensure that the chosen concentration has a reliable effect on the cells.
